# Artificial selection on storage protein 1 possibly contributes to increase of hatchability during silkworm domestication

**DOI:** 10.1371/journal.pgen.1007616

**Published:** 2019-01-22

**Authors:** Ya-Nan Zhu, Li-Zhi Wang, Cen-Cen Li, Yong Cui, Man Wang, Yong-Jian Lin, Ruo-Ping Zhao, Wen Wang, Hui Xiang

**Affiliations:** 1 Guangdong Provincial Key Laboratory of Insect Developmental Biology and Applied Technology, Institute of Insect Science and Technology, School of Life Sciences, South China Normal University, Guangzhou, China; 2 State Key Laboratory of Genetic Resources and Evolution, Kunming Institute of Zoology, Chinese Academy of Sciences, Kunming, China; 3 College of Life Sciences, Xinyang Normal University, Xinyang, China; 4 Center for Ecological and Environmental Sciences, Key Laboratory for Space Bioscience & Biotechnology, Northwestern Poly-technical University, Xi’an, China; New York University, UNITED STATES

## Abstract

Like other domesticates, the efficient utilization of nitrogen resources is also important for the only fully domesticated insect, the silkworm. Deciphering the way in which artificial selection acts on the silkworm genome to improve the utilization of nitrogen resources and to advance human-favored domestication traits, will provide clues from a unique insect model for understanding the general rules of Darwin's evolutionary theory on domestication. Storage proteins (SPs), which belong to a hemocyanin superfamily, basically serve as a source of amino acids and nitrogen during metamorphosis and reproduction in insects. In this study, through blast searching on the silkworm genome and further screening of the artificial selection signature on silkworm SPs, we discovered a candidate domestication gene, i.e., the methionine-rich storage protein 1 (SP1), which is clearly divergent from other storage proteins and exhibits increased expression in the ova of domestic silkworms. Knockout of *SP1* via the CRISPR/Cas9 technique resulted in a dramatic decrease in egg hatchability, without obvious impact on egg production, which was similar to the effect in the wild silkworm compared with the domestic type. Larval development and metamorphosis were not affected by *SP1* knockout. Comprehensive ova comparative transcriptomes indicated significant higher expression of genes encoding vitellogenin, chorions, and structural components in the extracellular matrix (ECM)-interaction pathway, enzymes in folate biosynthesis, and notably hormone synthesis in the domestic silkworm, compared to both the *SP1* mutant and the wild silkworm. Moreover, compared with the wild silkworms, the domestic one also showed generally up-regulated expression of genes enriched in the structural constituent of ribosome and amide, as well as peptide biosynthesis. This study exemplified a novel case in which artificial selection could act directly on nitrogen resource proteins, further affecting egg nutrients and eggshell formation possibly through a hormone signaling mediated regulatory network and the activation of ribosomes, resulting in improved biosynthesis and increased hatchability during domestication. These findings shed new light on both the understanding of artificial selection and silkworm breeding from the perspective of nitrogen and amino acid resources.

## Author summary

Like other domesticates, nitrogen resources are also important for the only fully domesticated insect, the silkworm. Deciphering the way in which artificial selection acts on the silkworm genome to improve the utilization of nitrogen resources, thereby advancing human-favored domestication traits, will provide clues from a unique insect model for understanding the general rules of Darwin's theory on artificial selection. However, the mechanisms of domestication in the silkworm remain largely unknown. In this study, we focused on one important nitrogen resource, the storage protein (SP). We discovered that the methionine-rich storage protein 1 (SP1), which is divergent from other SPs, is the only target of artificial selection. Based on functional evidence, together with key findings from the comprehensive comparative transcriptome, we propose that artificial selection favored higher expression of *SP1* in the domestic silkworm, which would influence the genes or pathways vital for egg development and eggshell formation. Artificial selection also consistently favored activated ribosome activities and improved amide and peptide biosynthesis in the ova, like what they may act in the silk gland to increase silk-cocoon yield. We highlighted a novel case in which artificial selection could directly act on a nitrogen resource protein associated with a human-desired domestication trait.

## Introduction

The silkworm, *Bombyx mori*, is the only fully domesticated insect species, originating from its wild ancestor, *B*. *mandarina*, approximately 5000 years ago. During the domestication process, the domestic silkworm evolved rapidly under human-preferred selection. Deciphering the way in which artificial selection acts on the silkworm genome to produce human-favored domestication traits will provide clues from a unique insect model for understanding Darwin's theory of artificial selection [1]. Recently, through genome-wide screening of selection signatures in a large batch of domestic and wild silkworms, we identified candidate domestication genes that enriched nitrogen and amino acid metabolism pathways, specifically in glutamate and aspartate metabolism. Knockout of two involved genes resulted in abnormal metamorphosis and decreased cocoon yield [2]. These findings suggest that, like domestic plants and animals, domestic silkworms also tend to have efficient utilization of nitrogen resources to adapt to human-preferences [2–4]. In addition to the glutamate and aspartate metabolism, which is an ammonia re-assimilated system [5], we further wonder whether other kinds of nitrogen supplies are also affected by artificial selection. If this is the case, how have they contributed to silkworm phenotypic changes during domestication?

Insect storage proteins (SPs) are another important resource of amino acids and nitrogen. Specifically, SPs are repositories of stored amino acids that belong to a special conserved arthropod hemocyanin superfamily [6]. Most insects have at least two main types of storage proteins, i.e., arylphorin and methionine-rich storage proteins; some species have other atypical SPs [7]. *SP*s have been cloned or predicted in many insect species, including Lepidoptera moths and butterflies [8–11]. Insect SPs are believed to serve as a source of amino acids and nitrogen for pupae and adults during metamorphosis and reproduction [12], however there is little solid functional evidence of their biological significance [10, 13]. In plants, storage proteins are mainly reserved in seeds, where, along with other nutrients such as oil and starch, they supply energy for seed germination and growth [14, 15]. Particularly in crops, seed SPs act to provide energy for humans and animals and thus are of great interest and a target for breeding and improvement [14–16].

In the domestic silkworm, previous studies preliminarily characterized the gene and protein expression patterns of four *SP*s [8, 17–19]. *SP1* is female-biased expressed when entering the last instar, and only accumulates in the female pupa [8, 20, 21]. It has been suggested that SP1 contributes to adult female characters and is related to the synthesis of vitellogenin (Vg), the precursor of yolk protein [17]. SP2 couples with SP3 to form a heterohexamer and has inhibitory effects on cell apoptosis [18, 19]. SSP2 was a heat resistant protein and suggested has a cell-protective function [19]. The determination as to whether or not SPs are also important in silkworm domestication, as is the case in domesticated plants, awaits a thorough exploration of their biological and evolutionary significance.

Development of genomics and genome-editing techniques provide tools for efficiently deciphering the evolutionary and functional significance of particular genes [22, 23]. In this study, we conducted a genome-wide identification of the silkworm SPs. Taking advantage of the genomic data resource of a batch of representative domestic and wild silkworms [2], we performed selection signature screening of the silkworm SPs followed by functional verification via the CRISPR/Cas9 knockout system and comprehensive comparative ova transcriptomes of wild-type and mutant silkworms as well as domestic and wild silkworms. Our findings suggest that artificial selection on *SP1* contributes to increased egg hatchability during silkworm domestication, possibly by promotion of vitellogenin, influence of hormone synthesis and egg development and eggshell formation. These results provide a novel case with functional evidence for the determination of a regulatory framework on a silkworm domestication gene, revealing that artificial selection acting on the nitrogen and amino acid supply is also required for improved silkworm reproduction.

## Results

### *SP1* is divergent from other *SPs* and is the only *SP* targeted by artificial selection

**Fig 1:**
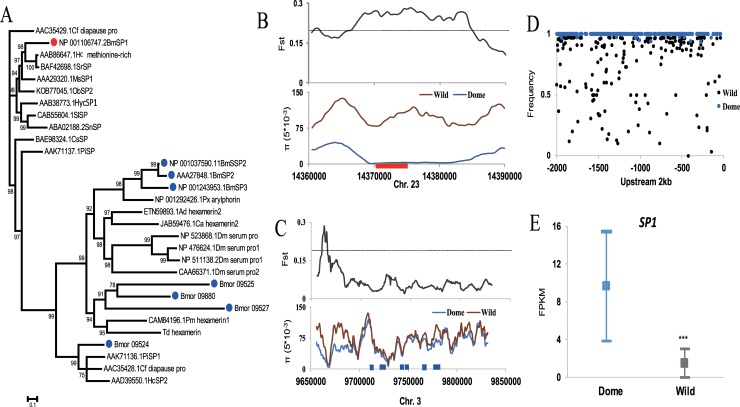
Molecular evolution of silkworm SPs. (A) Phylogenetic tree of insect Storage proteins based on Bayesian inference analyses. Full-length amino acid sequences were aligned to generate a phylogenetic tree. Bayesian posterior probability was shown for each node. Cf, *Choristoneura fumiferana*; Bm, *Bombyx mori*; Hc, *Hyalophora cecropia*; Sr, *Samia ricini*; Ms, *Manduca sexta*; Ob, *Operophtera brumata*; Hyc, *Hyphantria cunea*; Sl, *Spodoptera litura*; Sn, *Sesamia nonagrioides*; Cs, *Chilo suppressalis*; Pi, *Plodia interpunctella*; Px, P*lutella xylostella*; Ad, *Anopheles darlingi*; Ca, *Corethrella appendiculata*; Dm, *Drosophila melanogaster*; Pm, *Perla marginata*; Td, *Thermobia domestica*. Accession number for each protein is indicated ahead of abbrevation of each species. (B-C). Selection signatures of the silkworm *SP*s. Signature index—Population divergence coefficient (*Fst*) between the Chinese local trimoulting (CHN_L_M3) domestic silkworm group and the wild silkworms and nucleic acid diversity (*π*) in the silkworm populaton is shown along the genomic regions covering the *SP* genes. Dashed lines represent the top 5% *Fst* cutoff. The red square represents *SP1* region which is located in Chromosome 23 and the blue squares represents the other *SPs* which are clustered in Chromosome 3. (D) Plotting of frequency of reference genotype for each SNP position in the upstream 2 kb region of *SP1*, indicating many mutant alleles with fairly high allelic frequency in the wild silkworm population. (E) Expression level indicated as FPKM of *SP1* in the ova of domestic and wild silkworms. The three data pot indicate the highest value with 95% confidence, the average and the lowest value with 95% confidence, respectively. ***, FDR corrected *p* <0.001. Dome, the domestic silkworm. Wild, the wild silkworm.

In total, we identified 8 *SP*s in the silkworm genome by means of a blast search. Among which SP2 were not annotated in the gene list. Of these, SP1 exhibited the highest methionine content (10.98%) (Table 1). Phylogenetic analysis showed that *SP1* was located in one distinct clade, whereas other SPs were in another, indicating an obvious divergence between *SP1* and the remaining SPs (Fig 1A). *SP1* is located on chromosome 23 while the other SPs are clustered on chromosome 3, suggesting possible tandem duplication events during evolution. Interestingly, by screening artificial selection signatures on the genomic region bearing *SP1* and the other *SP*s respectively, we detected a strong selection signature in the *SP1* region of the domestic silkworm (see Material and methods), since there was notably reduced nucleotide diversity in the domestic silkworm group (Fig 1B and 1C). Furthermore, we detected strong differentiation in allelic frequency upstream of *SP1* (Fig 1D). Correspondingly, *SP1* was differentially expressed in the ova of domestic and wild silkworms, with higher expression in the domestic one (Fig 1E, S1 Fig). We also detected 11 SNPs that caused amino acid changes in the coding sequence of the gene (S2 Fig); the biological significance of these SNPs requires further evaluation. These results suggest that artificial selection acting on *SP1* during silkworm domestication may affect the function of this gene in domestic silkworms. At the very least, we can infer that selection may favor higher expression in the domestic silkworm.

**Table 1:** Basic information and methionine composition of silkworm SPs.

	Protein length (KD)	Protein weight (No. amino acid)	No. Met	Proportion of Met (%)
**Bmor_06909(SP1)**	87.25	747	82	10.98
**Bmor_09882(SSP2)**	83.45	703	23	3.27
**SP2**	83.46	704	27	3.84
**Bmor_09881(SP3)**	82.85	696	22	3.16
**Bmor_9524**	87.39	743	49	6.59
**Bmor_9525**	99.02	866	18	2.08
**Bmor_9527**	77.61	651	20	3.07
**Bmor_9880**	47.42	441	3	0.73

### Knockout of *SP1* by CRISPR/Cas9 system

**Fig 2:**
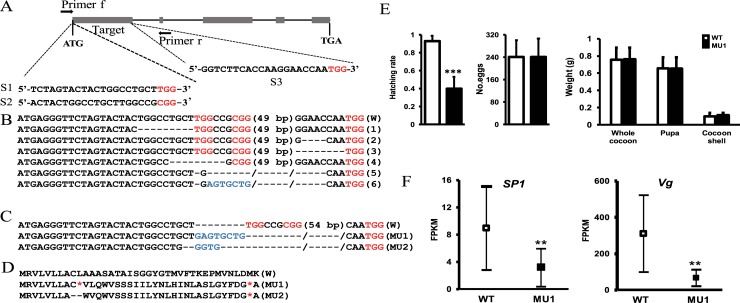
Cas9/sgRNA mediated gene editing of *SP1* in the silkworm. (A) Schematic diagram of sgRNA targeting sites. The five boxes indicate the five exons of *BmSp1*, and the black line with blocks represents the gene locus (blocks, exons; lines between blocks, introns). All sgRNA targeting sites are located on the first exon (S1, S2, and S3) and the PAM sequence is labeled in red. Sp1-F and Sp1-R were the primers used for mutation screening. (B) Various types of mutations in G0 injected embryos. Deletions are indicated by hyphens and insertions are shown in blue lowercase letters. The PAM sequence is in red. (C) Two types of mutations identified from homozygous mutant silkworms. W, wild type. MU1, *SP1* mutant type 1. There was an 8bp insertion followed by a 63 bp deletion in the *SP1* coding sequences; MU2, *SP1* mutant type 2. There was a 4 bp insertion followed by a 65 bp deletion. (D) Comparison of inferred amino acid sequences of homozygous mutant silkworms and the wild type. The missing amino acids are replaced with dashes. Premature stop codons are shown in red asterisk. (E) Phenotype assay of the mutants. Hatching rate of *SP1* mutants (MU1) decreased to about 51%. In total, 83 replicates of hatchings for MU1, 43 replicates for MU2 and 14 replicates for wild-type were used for statistical analysis. Number of egg produced, whole cocoon weight, pupa weight and cocoon layer weight, and pupa weight/whole cocoon weight ratio of mutants and wild-type silkworms, indicating no significant differences. In total, 32 replicates for mutants and 11 replicates for wild-type were used. Error bar: SD. (F) Expression level indicated as FPKM of *SP1* and *Vg* in the ova of domestic and wild silkworms. The three data pots indicate the highest value with 95% confidence, the average and the lowest value with 95% confidence, respectively. *, ** and *** represented significant differences at the 0.05, 0.01, 0.001 level (t-test).

To explore the possible phenotypic influence of artificial selection of *SP1* acting on the domestic silkworm, we first investigated the biological role of this gene in the silkworm using CRISPR/Cas9 knockout system. For the single guide RNA (sgRNA) design, we selected highly specific targets in the first exon, close to the translation starting site; namely, S1 and S2 (Fig 2A). We chose another site S3 close to the end of the first exon, more than 60 bp downstream from S1 and S2 (Fig 2A and Table 2) to obtain a potentially large fragment deletion by injecting the pool of three gRNAs. After mutation screening of the injected eggs (G0 generation), the gRNAs targeting the above three sites successfully guided DNA editing and generated a variety of mutation types, including 4–9 bp deletions or small insertions followed by a large deletion (Fig 2B).

**Table 2:** Primers used in this study.

Primer	Name	Primer
Sp1gRNA1-f	gaaattaatacgactcactataTCTAGTACTACTGGCCTGCTgttttagagctagaaatagc	Preparation of sgRNA templates
Sp1gRNA2-f	gaaattaatacgactcactataACTACTGGCCTGCTTGGCCGgttttagagctagaaatagc	Preparation of sgRNA templates
Sp1gRNA3-f	gaaattaatacgactcactataGGTCTTCACCAAGGAACCAAgttttagagctagaaatagc	Preparation of sgRNA templates
Sp1gRNA-r	AAAAGCACCGACTCGGTGCCACTTTTTCAAGTTGATAACGGACTAGCCTTATTTTAACTTGCTATTTCTAGCTCTAAAAC	Preparation of sgRNA templates
Sp1-F	CGGAAATATGGCCTATAATGCT	Identification of somatic mutations
Sp1-R	TGTCTACACGGATCATCACC	Identification of somatic mutations

Note: Sp1gRNA1-f, Sp1gRNA2-f, Sp1gRNA3-f and Sp1gRNA-r were used for amplification of DNA templates to generate gRNAs targeting S1, S2, S3 sites; Sp1-F and Sp1-R for detection of targeting sites.

Through screening of the exuviae of the fifth instar larvae in the G0 cocoons, we successfully identified 26 mosaic mutant G0 moths. We then generated pairwise crosses of those G0 mutants with similar mutant genotypes from the G1 populations. After mutation screening of the G1 eggs, we selected two populations with large deletions for further feeding and mutation screening (see Material and methods). Finally, in the G2 generation we obtained two types of homozygous mutants, i.e., MU1 and MU2 (Fig 2C). In MU1, there was an 8 bp insertion followed by a 63 bp deletion in the *SP1* coding sequences. In MU2, there was a 4 bp insertion followed by a 65 bp deletion. The mutations occurred at +29 and +26 bp of the first *SP1* exon in MU1 and MU2, respectively (Fig 2C), resulting in reading frame shift mutations and severe premature termination close to the translation starting site, with stop signals at +10 aa and +37 aa of the *SP1* protein (Fig 2D).

### Both the *SP1* mutant and the wild silkworm had decreased hatching rates and *Vg* expression

We selected and maintained the MU1 population for assay on phenotypes related to reproduction and metamorphosis, such as the number of eggs, hatching rate, pupa weight, and cocoon weight. Compared with the wild-type, which exhibited hatching rates of approximately 90%, the hatching rates of the *SP1* mutants were dramatically lower, with a mean value of about 40% (Fig 2E), although neither the number of eggs produced nor the whole pupa weight or cocoon shell weight were noticeably affected (Fig 2E). Given that the data were obtained from large replicates (83 replicates for the hatchability assay and 240 replicates for the pupa and cocoon weights), the results are robust. Loss-of-function mutation resulted in significantly decreased expression of *SP1* and *Vg* in the ova, based on the RNA-seq data (Fig 2F). These results suggest that in the silkworm *SP1* may positively affect the expression of ova *Vg* and contribute to silkworm egg development.

**Fig 3:**
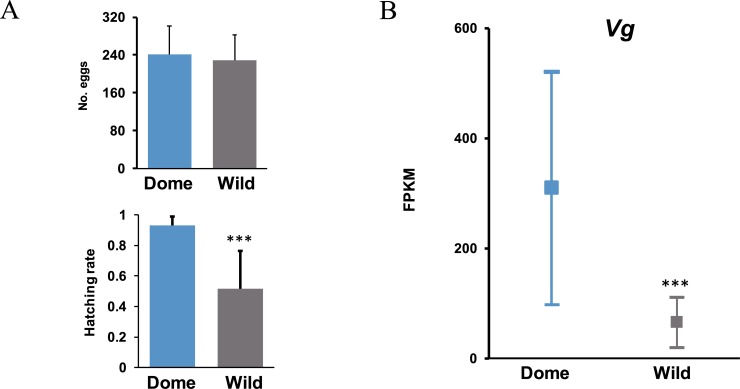
The artificial selection on *SP1* might improve silkworm hatching rate during domestication. (A) The egg production and hatching rates in the domestic group and wild silkworms. Error bar: SD. Wild_1 and Wild_2 indicated the results from two repeat times. (B) Expression level indicated as FPKM of *Vg* in the ova of domestic and wild silkworms. The three data pots indicate the highest value with 95% confidence, the average and the lowest value with 95% confidence, respectively. *, ** and *** represented significant differences at the 0.05, 0.01, 0.001 level.

Given that knockout of *SP1* resulted in a reduced hatching rate (Fig 2E) and that it is female-specific expressed in the pupa and adult stages, we suspect that it plays an important role in ova development, thus contributing to an efficient hatching process. During domestication, artificial selection preferred higher expression of *SP1*, thus may improve the silkworm hatching rate. As expected, we found that the hatching rate of the domestic silkworm was significantly higher than that of the wild one (Fig 3A). No obvious differences in egg production was detected between wild and domestic silkworms (Fig 3A). The lower hatching rate of the wild silkworm has also been reported in other studies [24, 25]. We further tested expression of *Vg* in the ova and discovered that consistently, it was significantly higher expressed in the domestic silkworm than in the wild silkworm (Fig 3B, S1 Fig). Promotion of *SP1* expression in the domestic silkworm thus results in the corresponding up-regulation of *Vg*, which further contributes to increased hatchability during silkworm domestication.

### Genes involved in egg development and eggshell formation were repressed both in SP1 mutant and the wild silkworm

**Fig 4:**
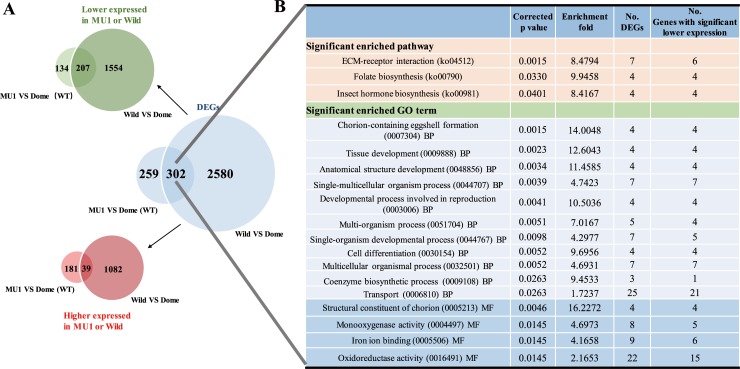
Differential expressed genes (DEGs) and functional enrichment analysis of the common genes from DEGs of two comparisons, i.e., from mutant VS the domestic silkwom wild type, and from the dometic and the wild silkworm. (A) Venn diagram of the DEGs between the wild type and the mutant, as well as domestic and wild silkworm (*Bombyx mandarina*). The blue Venn diagram represents the total DEGs, The green Venn diagram and the red Venn diagram represent significantly lower and higher expressed, respectively. (B) Functional enrichment analysis of common genes shared in the two sets of DEGs. Corrected *p*-value: *p*-value in hypergeometric test after FDR correction. All non-redundant terms that had corrected *p*-value < 0.05 are shown.

In order to further explore the regulation network and possible molecular mechanisms of female-specific *SP1* on egg hatchability, we generated comprehensive ova comparative transcriptome analyses between the wild-type and the mutant, as well as the domestic and wild silkworm (*Bombyx mandarina*), with 4.87~9.15 Gb RNA-seq data for each sample (S1 Table). We chose ova instead of fertilized eggs as target because silkworm *SP1* is female-biased expressed when entering the last instar and only accumulated in the female [8, 20, 21]. Comparative transcriptomics in this target tissue would directly focus mechanism of *SP1* on female reproductivity and avoid potential influence from the male. In total, there were 561 genes identified as differentially expressed genes (DEGs) in the *SP1* knockout mutants (MU1) compared to the wild-type silkworm, with significantly more down-regulated genes (341) than up-regulated (220) (*p* = 0.0003, Chi-squared test with Yates' continuity correction) (Fig 4A and S2 Table). As expected, we found many more DEGs (2882) between the wild and domestic silkworms, since wild silkworms are much more genetically and phenotypically different from the domestic one, compared with the silkworm mutant from the wild-type. It is interesting that in the 2882 DEGs, there were also significantly more lower expressed genes (1761) than higher expressed (1121) (*p* = 2.2e-16, Chi-squared test with Yates' continuity correction) (Fig 3A and S3 Table) in the wild silkworm. These results suggest that transcriptome repression in ova might be an output of *SP1* depletion in the *SP1* mutant (Fig 2F and Fig 4A) and a low expressional level of *SP1* in the wild silkworm (Fig 1E; Fig 4A and S1 Fig).

We identified 302 common genes in the two sets of DEGs. KEGG enrichment analysis indicated that these common DEGs were significantly enriched in pathways related to cell proliferation, such as ECM-receptor interaction and folate biosynthesis, as well as the hormone synthesis pathway, which is important in adult ovary development and female production [26, 27] (Fig 4B). Gene ontology (GO) enrichment analysis indicated that the common genes were enriched in reproduction related biological processes, such as chorion-containing eggshell formation (Fig 4B). These genes were also enriched in the molecular functions of the structural constituents of chorion (Fig 4B). In fact, they are all annotated as chorionic proteins, including 3 chorion class CB protein M5H4-like genes (*BGIBMGA009720*, *BGIBMGA009719*, *BGIBMGA009715*) as well as a chorion class B protein PC10 gene (*BGIBMGA009721*). All these chorion like genes showed significantly higher expression in the domestic silkworm, compared with both the mutant and the wild silkworm (Fig 4B, S2 Table, S3 Table). This pattern was also supported by Real-Time PCR validation in the domestic and wild silkworm (S1 Fig). Genes in ECM-receptor interaction pathway include collagens and integrins (S3 Fig) and those in folate biosynthesis include folylpolyglutamate synthase, which involves in 7,8-Dihydrofolate (DHF) and 5,6,7,8-Tetrahydrofolate (THF), substrates for subsequent one carbon pool mediated by folate (S4 Fig). The enriched hormone synthesis pathway includes genes functioning in both juvenile and molting hormones (S5 Fig). Extend to all the enriched genes, it is notable that most of these enriched genes were relatively highly expressed in the domestic silkworm, compared with *SP1* mutant and the wild silkworm (Fig 4B).

We further generated enrichment analyses on DEGs on the two sets of DEGs independently and observed consistent pattern (Tables 3 and 4). Functional enrichment analysis of DGEs between wild-type silkworm and *SP1* mutant silkworm revealed a significantly enriched the KEGG pathway “ECM-receptor interaction” as well as other three pathways with marginal significances, insect hormone biosynthesis, Glycine, serine and threonine metabolism and Folate biosynthesis (Table 3). The enriched GO terms included eggshell formation process and structural constituent of chorion. Most of the genes in these two GO terms were down-regulated in the mutant (Table 3). Consistently, These GO items were also in the top rank with the lowest *p* values when analyzing the DEGs between wild and domestic silkworm (Table 4). Nearly all of the genes involved in these KEGG and GO terms showed significant lower expression level in the wild silkworm, i.e., up-regulated in the domestic silkworm (Table 4). These results further supported that in the wild silkworm, low expression level of *SP1* may be associated with suppressed expression of genes in the eggshell formation process.

**Table 3:** Functional enrichment of differential expressed genes between the loss-of-function *SP1* mutants and the wild-type domestic silkworms.

ID	Description		Corrected *p* value	Enrichment fold	No. DEGs	No. down regulated genes in the mutants
ko04512	ECM-receptor interaction	0.02159		5.8202	7	6
ko00981	Insect hormone biosynthesis^a^	0.0615		6.1265	5	5
ko00260	Glycine, serine and threonine metabolism^a^	0.06443		3.9748	7	5
ko00790	Folate biosynthesis^a^	0.07961		6.6516	4	4
**Molecular Function**						
GO:0005213	Structural constituent of chorion	0.00008		13.1568	6	5
GO:0004497	Monooxygenase activity	0.00204		4.5294	14	4
GO:0016491	Oxidoreductase activity	0.00295		2.1087	39	21
GO:0005506	Iron ion binding	0.01655		3.4973	14	6
GO:0046906	Tetrapyrrole binding	0.01389		3.9471	10	5
GO:0005215	Transporter activity	0.04483		1.8916	30	24
GO:0004866	Endopeptidase inhibitor activity	0.04181		4.3856	6	3
GO:0004517	Nitric-oxide synthase activity	0.04753		19.7352	2	0
**Biological Process**						
GO:0051704	Multi-organism process	0.00000		10.8421	13	7
GO:0007292	Chorion-containing eggshell formation	0.00003		12.2321	6	5
GO:0043207	Response to external biotic stimulus	0.00003		10.7031	7	1
GO:0030154	Cell differentiation	0.00020		8.4684	6	5
GO:0003006	Developmental process involved in reproduction	0.00020		9.1741	6	5
GO:0002376	Immune system process	0.00045		7.7255	8	2
GO:0006810	Transport	0.00070		1.7666	44	32
GO:0019731	Antibacterial humoral response	0.00087		9.1741	5	0
GO:0044707	Single-multicellular organism process	0.00351		3.8403	9	7
GO:0006809	Nitric oxide biosynthetic process	0.02308		18.3481	2	0
GO:0018149	Peptide cross-linking	0.02308		18.3481	2	0
GO:0072593	Metal ion transport	0.03367		3.2932	7	0
GO:0006811	Ion transport	0.04042		1.9971	16	10

^a^ marginal significance.

**Table 4:** Functional enrichment of differential expressed genes between the wild and domestic silkworms.

ID	Description	Corrected *p* value	Enrichment fold	No. DEGs	No. up-regulated genes in the domestic silkworm
ko03010	Ribosome	0.00001	1.7870	50	49
**Molecular Function**					
GO:0003735	Structural constituent of ribosome	0.00003	1.7070	44	42
GO:0020037	Heme binding	0.02886	1.8963	26	24
**Biological Process**					
GO:0046434	Organophosphate catabolic process	0.35386	3.0761	6	4
GO:1901292	Nucleoside phosphate catabolic process	0.35386	2.9907	5	3
GO:1901361	Organic cyclic compound catabolic process	0.35386	2.4846	9	6
GO:0007304	Chorion-containing eggshell formation	0.35386	2.3925	6	5
GO:0019439	Aromatic compound catabolic process	0.35386	2.3925	8	5
GO:0044270	Cellular nitrogen compound catabolic process	0.35386	2.2838	7	5
GO:0006030	Chitin metabolic process	0.35386	1.8724	12	7
GO:0006022	Aminoglycan metabolic process	0.35386	1.8488	17	12
GO:1901564	Organonitrogen compound metabolic process	0.35386	1.1729	117	98

### Ribosome proteins and genes in amide and peptide biosynthetic processes were also repressed in the wild silkworm

DEGs between domestic and wild silkworms were significantly enriched in function of structural constituent of ribosome. The related genes are mostly ribosome proteins (S6 Fig). We also noted that the related biological processes, such as amide and peptide biosynthesis, was also in the top rank with the lowest *p* value (Table 4). The related genes were also up-regulated in the domestic silkworm. During domestication, there might be other factors that contribute to improved hatchability, such as, improved amide and peptide biosynthesis and activated ribosome activities in the ovaries.

## Discussion

Nitrogen resources are very important for silkworm domestication. The domestic silkworm tends to efficiently utilize nitrogen resources to yield protein outputs to adapt to human-preference, such as the economically important product, the cocoon. In this study, we discovered that artificial selection could directly act on a nitrogen resource gene, i.e, storage protein 1 (SP1), to improve silkworm hatchability. SPs are also target loci of breeding in crops [28]. However, with edible crops, human can directly benefit from the nutrients of these improved SPs [16], whereas in the silkworm, the SPs benefit is in the form of increased silkworm reproductive capacity.

Among all the *SP*s identified, *SP1* is quite divergent and somewhat unique from the others, both in terms of genomic location and phylogenetic position. A similar pattern was also observed in other Lepidoptera species, such as the tobacco hornworm, *Manduca sexta* [29], suggesting that *SP1* may have evolved independently, while the other types of *SPs* might have experienced duplication during Lepidoptera evolution. Methionine-rich SP1 seems to be of special interest, since methionine is reported to be an important amino acid in the trade-off between growth and reproduction [30]. In *Drosophila*, dietary methionine restriction extends lifespan [30], while in grasshoppers, a reduced reproduction-induced increase in expression methionine-rich protein occurred during life extension [31]. Similarly, in the beet armyworm, silencing of *SP1* by RNA interference (RNAi) decreases larval survival, which indicates the role of the methionine-rich SP in growth and metamorphosis[13]. We therefore added a new evidence that different to grasshopper [31] and the beet armyworm[13], but similar to *Drosophila*[30], silkworm methionine-rich SP1 functions in the reproduction process but does not obviously affect growth.

Given that in cocoon-producing silk moths, other nitrogen utilization system such as the glutamate /glutamine cycle, have been reported to be vital in metamorphosis silk-cocoon production [2, 5, 32], we suspect that the strategy of nitrogen resource allocation via storage proteins may have diverged or modified during Lepidoptera insect evolution. In the silkworm, the function of *SP1* is limited to influencing the egg hatching rate. Artificial selection acted only on *SP1* rather than other *SP*s, suggesting the importance of *SP1* for human-preferred domestication traits, i.e., increased hatchability.

Ova comparative transcriptome analyses further illustrated a framework of regulatory network of *SP1* on hatchability. Firstly, there are many genes near the bottom of the regulatory network, including vitellogenin(Vg), chorion proteins, structural component proteins in the extracellular matrix (ECM)-interaction pathway such as collagen and integrins, and synthetase in folate biosynthesis are all generally repressed in both the *SP1* mutant and the wild silkworm. Thus, artificial selection acts on *SP1* for increased hatchability, possibly associated with the influence of those genes, pathway or biological processes, and finally contributes to an improved performance of ovary. Vg is the main nutrient for silkworm egg formation and embryonic development[33]. It appears and accumulates at the stage when SP1 rapidly declines and disappears in the fat body, shortly before the emergence of the adult silkworm [17, 34]. *SP1* may supply amino acids for the synthesis of Vg, as previously reported in *Plutella xylostella* [35]. We therefore suspect that deficiency of SPs might directly trigger an as yet unknown regulatory pathway for the expression or synthesis of Vg. Chorion proteins are the major component of the silkworm eggshell and perform the essential function of protecting the embryo from external agents during development, while simultaneously allowing gas exchange for respiration. Eggshell (chorion) is constructed by the ovarian follicle cells. The follicle cell epithelium surrounds the developing oocyte and, in the absence of cell division, synthesizes a multilayer ECM [36]. Eggshell ECM was usually linked by integrins, a family of transmembrane receptor proteins to the cytoskeleton of the oocyte. Via a series of signal transductions, ECM-integrins function in oocyte movement, differentiation, and proliferation [36]. Integrins were reported to function in formation of actin arrays in the egg cortex [37] and they were also involved in tracheole morphogenesis which affects respiration [38]. Repression of these genes are directly associated with deficient development and function of the ovary. Loss of function of SP in the mutant or low expression level in the wild silkworm of *SP* might influence development and function of the ovary, further reducing the expression of *Vg* and chorion genes.

Secondly, folate is known to be important for human fetal development [39]. In insects, folate also plays an important roles in egg development, possibly promoting the biosynthesis of nucleic acids in the ovaries, and evoking mitoses in cells of the collicular epithelium [40–42].

Last and interestingly, we found that the hormone synthesis pathway was also repressed in response to SP1 deficiency. Recent advances in hormone signaling indicate that in the adult insect, juvenile (JH) and molting hormones may cooperate to promote *Vg* expression and oocyte development [27, 43, 44]. Therefore, hormone signaling pathway might function in the regulatory network of SP in these downstream genes, although there are still black boxes in the regulation connections of these genes, which require further in-depth experimental exploration.

Notably, increased hatchability during domestication may not be solely attributed to the increased expression of *SP1* and the associated downstream genes, given that artificial selection acts on hundreds of gene loci in the silkworm genome [2, 45] and that the ova comparative transcriptome between wild and domestic silkworms identified many more genes than that between *SP1* mutant and wild-type silkworm. We observed significantly enriched pathway and structural constituent of ribosome, the protein translation machinery and the biological processes involved in nitrogen metabolism and, are generally up-regulated in the domestic silkworm compared with the wild one (Table 4). These results again supported the importance of nitrogen and amino acids in silkworm domestication, not only for silkworm protein output [2], but also for productivity.

Similar to other domesticates, hatchability of silkworm eggs directly determines the quantity of offspring, and thus it is an important productivity trait for human to favorably select during domestication. Based on the above results and the discussion, we propose that artificial selection, favors higher expression of *SP1* in the domestic silkworm, which would subsequently up-regulate the genes or pathways vital for egg development and eggshell formation. On the other hand, artificial selection consistently favors activated ribosome activities and improved nitrogen metabolism in the ova, as it might act in the silk gland for increased silk-cocoon yield [2]. In result, the domestic silkworm demonstrates improved egg hatchability compared with it wild ancestor.

## Materials and methods

### Silkworm strains

A multivoltine silkworm strain, Nistari, was used in all experiments. Larvae were reared on fresh mulberry leaves under standard conditions at 25°C. The wild silkworms were collected in Zhejiang Province, China and maintained as laboratory population in our lab.

### Silkworm genomic data resources

The genomic single nuclear polymorphic data (SNP) file (the VCF) for the domestic and wild silkworm obtained from DEYAD platform (https://doi.org/10.5061/dryad.fn82qp6) [2].

Reference genome and the annotation file used for RNA-seq data mapping were obtained from the Ensemble database (http://metazoa.ensembl.org/Bombyx_mori/Info/Index).

### Genomic search of *B*. *mori* SPs and phylogenetic analysis of SPs

The reference sequences of *B*. *mori* storage proteins (SP1, SP2, SSP2 and SP3) were retrieved from the NCBI GenBank. These sequences were used as query, searching for homologs in the *B*. *mori* genome by tblastn with e-value <10^−7^. Other insect homologs of the silkworm SPs were searched in GenBank (https://blast.ncbi.nlm.nih.gov/) by BLASTP with an e-value <10^−7^. We selected sequences from several representative Lepidoptera species and *Drosophila melanogaster* as candidate proteins for further analyses. The sequences of the SP1 homologs were aligned using MEGA 6.0 software [46]. A gene tree was constructed using MrBayes-3.1.2 with GTR + gamma substitution model [47]. The gene-ration number was set as 1000000 and the first 25% was set as burn-in. Other parameters were set as default.

### Molecular selection of *SP1* in domesticated and wild silkworm populations

Based on the available whole genomic single nuclear polymorphic data (SNP) of domesticated and wild silkworm populations [2], (https://doi.org/10.5061/dryad.fn82qp6), we screened the selection signatures of the silkworm *SPs*, according to Xiang et al’s pipeline [2]. Specifically, data from 19 samples of the early domesticated group (i.e., trimoulting local strains [CHN_L_M3]) of the domestic silkworm *Bombyx mori* and 18 samples of wild silkworm *B*. *mandarina* were used. The SNP data of the two chromosomes that *SP1* (Chromosome 23) and the cluster of the other *SPs* (Chromosome 3) were located were used to screen for the domestication signature. Chr. 23 is 20,083,478 bp in length and 2,046,397 SNPs were identified. Chr. 3 is 14,662,804 bp in length and 1,448,852 SNPs were identified, based on the published data. Allelic frequency and SNP annotation were calculated using in-house Perl scripts.

For the detection of selection signature during silkworm domestication, we set a very stringent threshold to screen out regions significantly deviated from the overall distribution. We only used windows within the top 1% of selective signatures (the corresponding *p* value of a Z test < 0.001) and applied *Fst* (fixation index) between the two groups to represent the selective signatures, taking the highest 1% value as the cutoff. The selection in the domestic silkworm group (i.e., the early domesticated group) was further confirmed by limiting *π* at a relatively low level (the lowest 5%).

### Design of sgRNA target and in vitro synthesis of sgRNA and Cas9 mRNA

The 20 bp sgRNA targets immediately upstream of PAM were designed by the online platform CRISPRdirect (http://criSpr.dbcls.jp/) [48]. The sgRNA DNA template was synthesized by PCR, with Q5 High-Fidelity DNA Polymerase (NEB, USA). The PCR conditions were 98°C for 2 min, 35 cycles of 94°C for 10 s, 60°C for 30 s, and 72°C for 30 min, followed by a final extension period of 72°C for 7 min. The sgRNA were synthesized based on the DNA template *in vitro* using a MAXIscript T7 kit (Ambion, Austin, TX, USA) according to the manufacturer’s instructions. The Cas9 construct was a kind gift provided by the Shanghai Institute of Plant Physiology and Ecology (Shanghai, China). The Cas9 vector was pre-linearized with the NotI-HF restriction enzyme (NEB, USA). The Cas9 mRNA was synthesized *in vitro* with a mMESSAGE mMACHINE T7 kit (Ambion, Austin, TX, USA) according to the manufacturer’s instructions. All related primers are shown in Table 2.

### Microinjection of Cas9/sgRNAs

Fertilized eggs were collected within 1 h after oviposition and microinjection was within 4 h. The Cas9-coding mRNA (500 ng/μL) and total gRNAs (500 ng/μL) were mixed and injected into the preblastoderm Nistari embryos (about 8 nl/egg) using a micro-injector (FemtoJet, Germany), according to standard protocols (Tamura, 2007). The injected eggs were then incubated at 25°C for 9–10 d until hatching.

### Cas9/sgRNA-mediated mutation screening and analyses of germline mutation frequency

To calculate the effect of Cas9/sgRNA-mediated gene mutation in the injected generation (G0), we collected ~10% of the eggs (64 out of 600) 5 d after injection to extract genomic DNA for PCR, with primers Sp1-F and Sp1-R (Table 2). The amplified fragments were cloned into a pMD19-T simple vector (Takara, Japan) and sequenced to determine mutation type.

When the injected G0 silkworms pupated, we collected silkworm exuviae from fifth instar larvae in each cocoon. Genomic DNA was extracted using a TIANamp Blood DNA Kit (Tiangen Biotech, Beijing) according to the manufacturer’s instructions. Individual mutation screening was generated with PCR at 94°C for 2 min, 35 cycles of 94°C for 30 s, 57°C for 30 s, and 72°C for 45 s, followed by a final extension period of 72°C for 5 min. The PCR products were cloned into the pMD19-T simple vector (Takara, Japan) and sequenced.

### Homozygous mutant screening and mutation effects on inferred protein

Mosaic mutant moths were obtained from the above mutation screening of exuviae DNA from fifth instar larvae. Moths with the same mutation site were pairwise crossed with each other to acquire G1 offspring. About 7 d after the G1 eggs were laid, we collected ~30 eggs from each offspring population from one parental pair and pooled them to extract genomic DNA for mutation screening by PCR. The amplified fragments were cloned into a pMD19-T simple vector (Takara, Japan) and sequenced to determine the exact mutation type. Two G1 offspring populations with large deletions in *BmSp1* were selected for further breeding. At the pupa stage, 20 randomly selected individuals within each population were subjected to mutation screening of exuviae DNA. Homozygous mutant moths with the same identified mutant genotype were crossed to acquire G2 offspring. Mutation effects on proteins were evaluated using MEGA 6/0 software[46] through codon alignment of the wild-type and the mutant.

### Phenotypic assay

On the fourth day of pupation (P4), we weighed and recorded the whole cocoon weight, pupa weight, and cocoon shell weight. In total, data from 240 SP1-MU1 mutants and 110 wild-type silkworms were recorded respectively.

Offspring of the homozygous mutants and wild-type silkworms were incubated at 25°C for 9–10 d until hatching. The number of eggs produced and hatched from each female moth were recorded respectively. The egg hatching rates were then determined. Eighty-three replicates were set for the *SP1* mutant and 17 for the wild-type populations, respectively. These assays were also generated for 20 wild silkworms.

Student’s *t*-test was used to analyze the significance of the differences. For comparisons of datasets with unbalanced size, Student’s *t*-test with FDR (false discovery rate) correction was used. Specifically, for the cocoon- and pupal- related traits, we divided the samples of *SP1* mutant to two groups, consisting of 120 samples for each, and generated *t*-test with the wild type respectively. The average *p* value followed by FDR correction was used to verify the significance.

As for the analysis of the number of eggs and hatching rates, we divided the samples of *SP1* mutant to 4 groups, consisting of 20 or 21 samples for each, and generated *t*-test with the comparable data from the wild-type silkworms, respectively. Average *p* value followed by FDR correction was used to verify the significance.

### Ova dissection, total RNA isolation and RNA-seq

Ova from newly emerged virgin moth of the domestic wild type silkworm, SP1 mutant and the wild silkworm were dissected and collected for RNA extraction with three replicates set for each. Total RNA were isolated using TRIzol (Invitrogen). For each sample, RNA were sent to Novogene Bioinformatics Institute (Beijing, China) for cDNA library construction and RNA-seq by Illumina Hiseq 2500 (Illumina, San Diego, CA, USA) with 125 bp paired-end reads according to the manufacturer’s instructions.

### Analyses on RNA-seq data

Raw data were filtered with the following criteria: (1) reads with ≥ 10% unidentified nucleotides (N); (2) reads with > 10 nt aligned to the adapter, allowing ≤ 10% mismatches; and (3) reads with > 50% bases having phred quality < 5. The clean data were mapped to the *Bombyx mori* reference genome using Tophat with 2 nt fault tolerance and analyzed using Cufflinks [49]. The relative expression value of each gene was calculated using the widely used approach, i.e., fragments per kilobase of exon per million pair-end reads mapped (FPKM) [49], using Cuffdiff In order to identify differentially expressed genes (DEGs), Cuffdiff was further used to perform pairwise comparisons between wild-typed and SP1 mutant samples, as well as the wild and domestic silkworm, respectively, with corrected *P*-value of 0.05 <5 and Log2-fold change>1.

KEGG and GO enrichment analyses of DEGs were performed with an online platform (http://www.omicshare.com/tools/), using all the expressed genes (FPKM >1) in the ova of virgin moth of *Bombyx mori* as background.

### Real-time PCR validation

We used real-time PCR to evaluate the results of RNA-seq data. The Ova of the domestic wild type silkworm and the wild type were dissected from newly emerged virgin moths. Total RNA was digested with DNase I (Takara) to remove the remaining DNA. For Complimentary DNA synthesis, 1ug of total RNA was used in the ReverAid First Strand cDNA Synthesis kit (Takara). Primers for real-time PCR were as follows: 5′ -GGCTTCACTGTCACCAGCACTT-3′ (BGIBMGA009715_f) and 5′ -ACCACAGCCGTAAGACACCAGA-3′ (BGIBMGA009715_r) for BGIBMGA009715; 5′ -GGGCTTATGATGCCGTAGGA-3′ (BGIBMGA009719_f) and 5′ -CGGTGGGAGTTATTGGTGATGT-3′ (BGIBMGA009719_r) for BGIBMGA009719; 5′ -ACCAGCATATCACCAATAGCACC-3′ (BGIBMGA009720_f) and 5′ -ATCGCCGCAGCCATACAGAA-3′ (BGIBMGA009720_r) for BGIBMGA009720; 5′ -GGCTTCATCTATCATCGCTCCAC-3′ (BGIBMGA009721_f) and 5′ -GCCACACCCATACGCCACTTCT-3′ (BGIBMGA009721_r) for BGIBMGA009721; 5′ -GGCAATTATAGCCGCCGTGTCC-3′ (Vg_f) and 5′ -GGCCAGGACTCTTTACCCGGAT-3′ (Vg_r) for Vg; 5′ -GACTCGTCGTGTAATGGAAAGC -3′ (SP1_f) and 5′ -ATGTGGGCAAGAGCATACCG -3′ (SP1_r) for SP1 and 5′ -CAGGCGGTTCAAGGGTCAATAC-3′ (RP49_f) and 5′-TACGGAATCCATTTGGGAGCAT-3′ (RP49_r) for the internal control, the ribosomal protein 49 (*Bmrp*49, AB48205.1). Real-time PCR was performed in three duplicates with SYBR Green PCR Mix (Bio-Rad) and subjected to the Roche LightCycler 480 Real-Time PCR System. The messenger RNA quantity of each gene was calculated with the 2-ΔΔCT method and normalized to the abundance of *RP49*.

## Supporting information

S1 Fig Real time-PCR validation on expression of some selected genes with differential expression between the domestic and wild silkworm. (EPS)

S2 Fig SNPs between the wild and domestic silkworm groups that cause non- synonymous mutations of *SP1*. (A). Amino acid alignment of SP1 protein of the domestic silkworm (*B*. *mori*) and the deduced SP1 protein sequence of the wild silkworm (*B*. *mandarina*) by inferred SNP data. (B). Allelic frequencies of the 11 non- synonymous mutations in the wild (the left column) and domestic silkworm (the right column). Blue, reference allele; Black, alternative allele. The 11 mutations of amino acid were labelled with numerals in red.
(EPS)

S3 Fig Scheme of the ECM-receptor interaction pathway. The shared DEGs of ova in two sets of comparison (*SP1* mutant vs wild type and wild vs domestic silkworm) are indicated by red frames.
(EPS)

S4 Fig Scheme of the folate biosynthesis pathway. The shared DEGs of ova in two sets of comparison (*SP1* mutant vs wild type and wild vs domestic silkworm) are indicated by red frames.
(EPS)

S5 Fig Scheme of the hormone biosynthesis pathway. The shared DEGs of ova in two sets of comparison (*SP1* mutant vs wild type and wild vs domestic silkworm) are indicated by red frames.
(EPS)

S6 Fig Scheme of the Ribosome pathway. The DEGs of ova between the wild and domestic silkworm are indicated by red frames.
(EPS)

S1 Table Summary of RNA-seq data. (XLSX)

S2 Table Information of the differentially expressed genes between *SP1* mutant (MU1) and the wild type (WT) domestic silkworm. (XLSX)

S3 Table Information of the differentially expressed genes between the wild and the domestic (Dome) silkworms. (XLSX)
